# A prospective observational study on the feasibility of subumbilical laparoscopic procedures under epidural anesthesia in sedated spontaneously breathing infants with a natural airway

**DOI:** 10.1111/pan.14302

**Published:** 2021-10-08

**Authors:** Philipp Opfermann, Peter Marhofer, Alexander Springer, Martin Metzelder, Markus Zadrazil, Werner Schmid

**Affiliations:** ^1^ Department of Anesthesia, General Intensive Care Medicine and Pain Therapy Medical University of Vienna Vienna Austria; ^2^ Department of Anesthesia and Intensive Care Medicine Orthopaedic Hospital Speising Vienna Austria; ^3^ Department of Surgery Division of Pediatric Surgery Medical University of Vienna Vienna Austria

**Keywords:** anesthesia, caudal, anesthesia, epidural, cryptorchidism, infant, laparoscopy, orchidopexy

## Abstract

**Background:**

Laparoscopic procedures are usually performed under general anesthesia with a secured airway including endotracheal intubation or supraglottic airways.

**Aims:**

This is a prospective study of the feasibility of subumbilical laparoscopic procedures under epidural anesthesia in sedated, spontaneous breathing infants with a natural airway.

**Methods:**

We consecutively enrolled 20 children <3 years old with nonpalpable testes scheduled for diagnostic laparoscopy with or without an ensuing orchidopexy, inguinal revision, or Fowler‐Stephens maneuver. Inhalational induction for venous access was followed by sedation with propofol and ultrasound‐guided single‐shot epidural anesthesia via the caudal or thoracolumbar approach using 1.0 or 0.5 ml kg^−1^ ropivacaine 0.38%, respectively. The primary outcome measure was block success, defined as no increase in heart rate by >15% or other indicators of pain upon skin incision.

**Results:**

Of the 20 children (median age: 10 months; IQR: 8.3−12), 17 (85%) were anesthetized through a caudal and 3 (15%) through a direct thoracolumbar epidural, 18 (90%) underwent a surgical procedure and 2 (10%) diagnostic laparoscopy only. Five patients (25%) received block augmentation using an intravenous bolus of fentanyl (median dose: 0.9 µg kg^−1^; IQR: 0.8−0.95) after the initial prick test and before skin incision. There was no additional need for systemic pain therapy in the operating theater or recovery room. No events of respiratory failure or aspiration were observed.

**Conclusions:**

In experienced hands, given our success rate of 100%, epidural anesthesia performed in sedated spontaneously breathing infants with a natural airway can be an alternative strategy for subumbilical laparoscopic procedures.

**What is already known**

Epidural anesthesia under sedation is a feasible method of anesthetic management for subumbilical procedures in infants.

**What this article adds**

Based on the data of twenty consecutive cases, epidural anesthesia under sedation with a natural airway can be considered as a potential management strategy in infants undergoing a subumbilical laparoscopic procedure.

## 1 INTRODUCTION

The standard management of pediatric laparoscopy is still by general anesthesia (GA) in the most centers. The rationale being to prevent aspiration and respiratory problems caused by the capnoperitoneum and associated increased abdominal pressure. An epidural alone approach is usually reserved to patients with specific conditions like lung or heart disease in whom GA poses a high risk.^1^

However, Krishnan et al recently published a retrospective study on caudal anesthesia and sedation without airway instrumentation as an alternative technique for the management of pediatric patients undergoing laparoscopic inguinal hernia repair.^2^

Common critical incidents associated with pediatric GA are respiratory events.^3^ They are partly caused by the relatively narrow airway in the youngest patients.^4^, ^5^, ^6^, ^7^, ^8^ An anesthetic approach avoiding airway instrumentation could be an advantageous management option to avoid intubation and its possible postoperative complications, particularly in ex‐preterm babies with a higher risk for apnea episodes.^9^ Neuraxial anesthesia could be one way to minimize the dose of anesthetic drugs. As no prospectively collected data were yet available referring to neuraxial anesthesia under sedation without airway “protection” for the management of children undergoing subumbilical laparoscopic surgery, we designed a prospective study using diagnostic laparoscopy for a nonpalpable testis in male infants as a model.

The primary endpoint of the study was the success rate of caudal or epidural blockade in sedated infants with a natural airway undergoing subumbilical laparoscopic surgery. The secondary endpoints included the amount of fentanyl and propofol needed during the procedures, and the total amount of analgesics administered in the recovery room.

## 2 MATERIAL AND METHODS

### 2.1 Preparations, patient enrollment, and exclusion criteria

This single‐center trial, designed in line with the STROBE statement, was approved by the institutional review board (Ethics Commission at Medical University of Vienna; ref. 1133/2017; approved on 24‐Aug‐2017) and entered into the German Clinical Trial Register (DRKS00012683; approved on 15‐Jul‐2019). The study was performed between July 21, 2019 and July 31,2020. All parents or legal guardians gave their written informed consent based on comprehensive information provided about the nature and scope of the study and the procedures to be conducted. We enrolled infants between 0 and 32 months old with nonpalpable testes scheduled for diagnostic laparoscopy with or without ensuing orchidopexy, inguinal revision with testicular remnant removal, or Fowler‐Stephens maneuver.

Exclusion criteria were contraindications to epidural anesthesia or amino‐amide local anesthetics, allergies, neurological disorders, local infection at the intended injection site, spine malformation, participation in another clinical study within 4 weeks before surgery, and clinically relevant ECG abnormalities such as AV block or bradycardia.

### 2.2 Patient preparation and premedication

**FIGURE 1:**
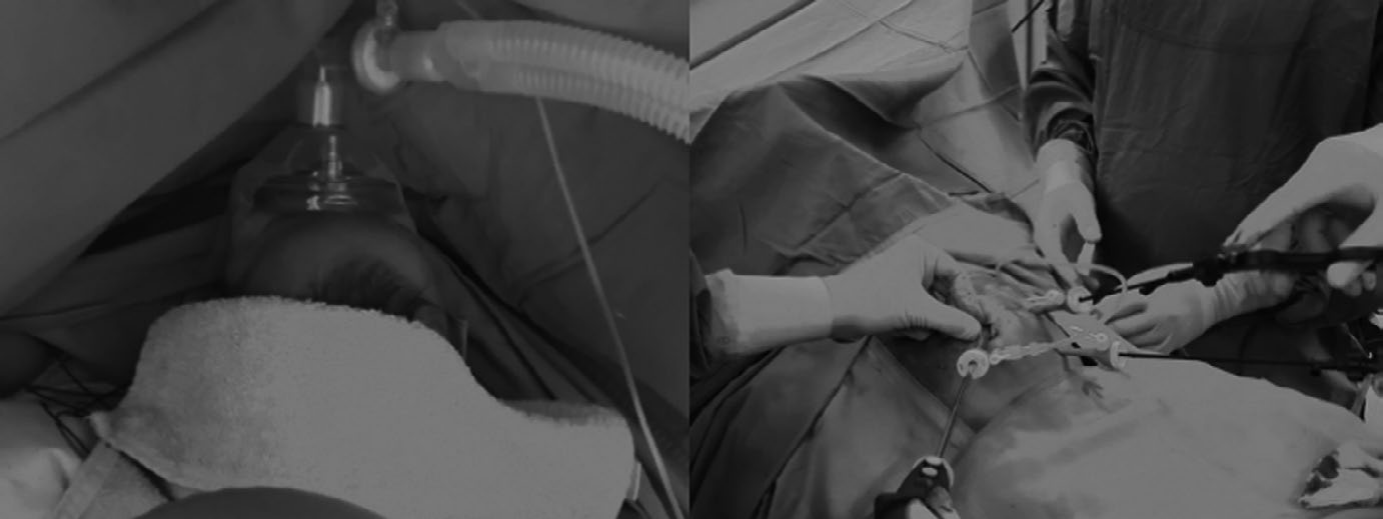
Intraoperative setting illustrating a sedated, spontaneously breathing patient with oxygen supplied through a face mask

Preoperative fasting conformed to our departmental standard of care (6 h for solid food, 4 h for breast milk, and 1 h for clear fluids). One hour before anesthesia induction, lidocaine 2.5% mixed with prilocaine 2.5% (EMLA 5% Cream; Astra Zeneca, Vienna, Austria) was applied to establish an intravenous access and on the foreseen puncture site in the spine area. Thirty minutes later, midazolam (Dormicum™; Roche, Vienna, Austria) 0.5 mg kg^−1^ was administered, not exceeding 15 mg. All patients ≤12 months of age received premedication with midazolam via a rectal application. The patients >12 months received oral premedication with flavored midazolam syrup. In the operating theater, cardiorespiratory monitoring was started with the child placed on a forced‐air warming blanket (Bair Hugger; Arizant, Eden Prairie, MN, USA). A gastric tube was placed for gentle aspiration of gastric juice, and anesthesia was induced with sevoflurane via face mask to establish a vascular access. Subsequently, the inhalation induction was stopped and propofol boli (up to 2 mg kg^−1^) were administered—if necessary—to facilitate caudal or epidural puncture. Standard monitoring included ECG, noninvasive arterial pressure, and peripheral oxygen saturation (SpO_2_), and, depending on age, the children received an infusion of either 10 ml kg^−1^ h^−1^ Elo‐Paed balanced (children <1 year) or 10 ml kg^−1^ h^−1^ Elo‐Mel isotone (Fresenius Kabi, Graz, Austria). Spontaneous breathing was continuously verified by an end‐tidal CO_2_ line fitted and attached with tape to a face mask, through which oxygen‐enriched air (FiO_2_: 0.40) was administered (Figure 1).

### 2.3 Single‐shot caudal or epidural anesthesia

For the single‐shot caudal or epidural anesthesia, the child was turned to a left lateral position with the hips and knees flexed. The particular techniques for single‐shot caudal and epidural anesthesia have been described elsewhere in detail.^10^, ^11^, ^12^ In short form, caudal blocks were performed using an ultrasound controlled “immobile needle technique” injecting 1.0 ml kg^−1^ ropivacaine 3.8 mg ml^−1^ into the epidural space. Epidural anesthesia was performed under ultrasound guidance at the level of the thoracolumbar transition (Th12‐L1) and using the loss of resistance with saline technique before injecting 0.5 ml kg^−1^ ropivacaine 3.8 mg ml^−1^ into the epidural space.

### 2.4 Postepidural preparations, laparoscopic technique, and surgical techniques

After placing the children in the required position for surgery, sterile preparation of the surgical area was performed, and a propofol infusion with a dose of 5 mg kg^−1^ h^−1^ was started. The rate of propofol was lowered or stopped as necessary or when the shortness of surgical procedure allowed doing so. The depth of sedation was considered adequate, when the patient was unconscious and arousable only with significant physical stimulation.

The cryptorchidism‐related laparoscopies were performed using a 5 mm infraumbilical or umbilical port for the camera and two more 5 mm ports in the left and right iliac fossa, respectively, to facilitate dissection. Peak intra‐abdominal pressure was limited to 10 mmHg. Specific laparoscopic findings were followed by specific surgical procedures: vas and testicular vessels entering the deep inguinal ring by groin exploration and single‐stage orchidopexy with fixation of the testis in a subdartos pouch; testicular remnant by excision of the testis (also considering fixation of the contralateral testis); and intra‐abdominal testis by first/second‐stage Fowler‐Stephens maneuvers.^13^

### 2.5 Evaluation of anesthesia, block success, and emergency management

Given an onset time of surgical analgesia of usually 10–15 min,^14^ the surgeon conducted a skin‐prick test after 10 min by applying forceps to the right and left subumbilical quadrants. Any resultant movement or increase in heart rate by >15% from baseline, suggesting inadequate blockade (eg, Wedensky block), was followed by an intravenous bolus of fentanyl at the discretion of the anesthesiologist in a dosage of up to 1 µg kg^−1^ and another 5 min of delay until skin incision. Blockade was considered successful in the absence of an increase in heart rate by >15% from baseline and any other signs of pain like tachypnea or movements upon skin incision.

A defined protocol of sequential management was defined for potential events of unsuccessful blockade: careful bag‐mask ventilation with <10 mmHg of inspiratory pressure, followed by our departmental standard of rapid‐sequence induction using propofol 2–3 mg kg^−1^, rocuronium 0.6 mg kg^−1^, and fentanyl 1 µg kg^−1^. Bradycardia or hypotension to >25% below baseline was treated by atropine 0.01 mg kg^−1^ or a fluid bolus of 10 ml kg^−1^, respectively. Equipment for advanced airway management was on standby and respiratory failure necessitating its use defined as paradoxal ventilation, disappearance of the end‐tidal CO_2_ curve, or drop in SpO_2_ to <92%.

### 2.6 Postoperative management in the recovery room

Pain scores were obtained on admission to the recovery room and every 30 min during the first two postoperative hours. They were based on the “modified objective pain scale” (OPS) with the objective behavioral parameters of crying, facial expression, position of torso, position of legs, and motor restlessness.^15^

Each of these was rated on a three‐point scale (0 = none; 1 = moderate; and 2 = severe). Given a maximum total score of 10, any two consecutive scores of ≥4 were followed up by i.v. administration of metamizol 10 mg kg^−1^ (Novalgin^®^, Sanofi‐Aventis GmbH, Frankfurt am Main, Germany) or nalbuphine 0.1 mg kg^−1^ (Nalbuphin Amomed^®^, Amomed Pharma GmbH, Vienna, Austria) depending on postoperative pain‐scoring. A clinical examination for local infection at the puncture site as well as for lower‐limb sensory and motor function was performed 24 h after surgery.

### 2.7 Study endpoints and statistical analysis

The primary endpoint of the study was the success rate of caudal or epidural blockade based on the aforementioned criteria of block success (see corresponding subheading above). Secondary endpoints included the amount of fentanyl and propofol needed during the procedures and the total amount of analgesics administered postoperatively in the recovery room. Patient characteristics are presented in the form of conventional summary statistics as median values with interquartile ranges (IQR) or as absolute numbers in conjunction with percentages. All data were screened for completeness, consistency, and outliers prior to statistical evaluation. Statistical software (IBM® SPSS®, version 24.0.0.0; IBM, Armonk, NY, USA) was used for all operations in this analysis.

## 3 RESULTS

### 3.1 Evaluable patients and surgical procedures

**FIGURE 2:**
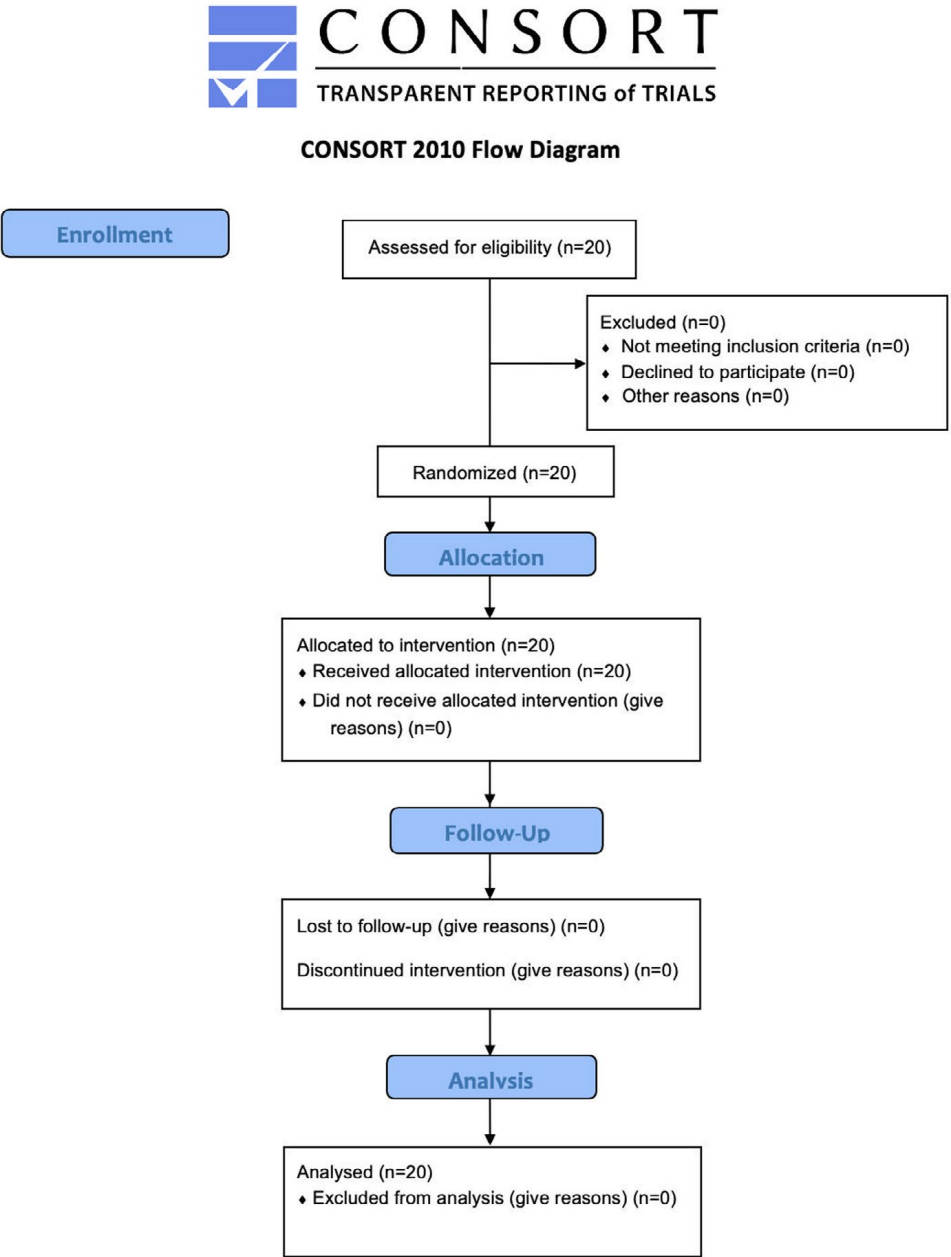
Flow chart of the study

A total of 20 consecutive pediatric patients with undescended testes who were scheduled for diagnostic laparoscopy with or without orchidopexy, first/second‐stage Fowler‐Stephens maneuver, or inguinal revision with testicular remnant removal were included in accordance with the inclusion and exclusion criteria (see Methods). Pertinent demographic data of this population are summarized in Table 1. As apparent from the flow chart in Figure 2, all of these 20 patients completed the study and could be evaluated. Table 2 provides an overview of the surgical procedures performed. Eighteen patients (90%) underwent a combined diagnostic and surgical procedure.

**TABLE 1:** Demographic and treatment‐related data (median values and interquartile ranges)

Age (months)	10	8.3−12
Weight (kg)	9.5	8.1−10
Height (cm)	74	71−78
Duration of surgical procedure (min)	39	31−61
Duration of capnoperitoneum (min)	19.5	4.3−49
Peak intra‐abdominal pressure (mmHg)	8	6.3−8.0
Total volume of ropivacaine 0.38% (ml)	12	9.3−15
Dose of fentanyl i.v. (µg kg^−1^)^a^	0.9	0.8−0.95
Total dose of propofol (mg kg^−1^) (boli and continuously infused propofol)	4.4	2.2–5.4

^a^ Five of the 20 procedures were delayed by 5 min for block augmentation using an intravenous bolus of fentanyl.

**TABLE 2:** Laparoscopic and surgical procedures performed (*n* = 20)

Laparoscopy for diagnosis only	*n* = 2	10%
Laparoscopy and orchidopexy	*n* = 3	15%
Laparoscopy and inguinal revision with remnant removal	*n* = 9	45%
Laparoscopy and laparoscopic remnant removal	*n* = 1	5%
Laparoscopy and 1st‐stage Fowler‐Stephens procedure	*n* = 4	20%
Laparoscopy and 2nd‐stage Fowler‐Stephens procedure	*n* = 1	5%

### 3.2 Block success, augmentation requirements, and dosage of sedation

All patients underwent a inhalation induction with sevoflurane until establishment of an IV access. Subsequently, nine patients got a bolus of propofol with a median dosage of 10 mg (IQR: 10−11). In 17 patients, the continuous propofol infusion was started after positioning the patient for surgery. The median total dose of propofol (Boli + continuously infused propofol) was 4.0 mg kg^−1^ (IQR: 2.2−5.4).

The success rate of the ultrasound‐guided epidural or caudal blocks performed with sedation was 100%. Of the 20 children, 17 (85%) were managed via a caudal block and 3 (15%) via thoracolumbar single‐shot epidural anesthesia. None of these cases exhibited any kind of respiratory failure or aspiration requiring mask ventilation or intubation for sequential airway management. Peripheral oxygen saturation remained stable between 97% and 100% throughout anesthesia and the diagnostic laparoscopies with or without surgery.

Five children (25%) required an additional intravenous bolus of fentanyl 0.9 µg kg^−1^ (IQR: 0.8−0.95) for the block to be effective, entailing another delay of 5 min after the initial skin‐prick test.

### 3.3 Postoperative pain scores and final examinations

The median modified OPS (IQR) values were 0 (0–1). Maximum value was 3 in two patients. Since all OPS scores remained below 4 in the recovery room, there was no need to provide any additional systemic pain therapy in any of the children. All examinations that were performed 24 h after surgery, including the neuraxial puncture sites as well as clinical assessments of sensory and motor function, led to normal findings.

## 4 DISCUSSION

This study presents a method of anesthesia management for diagnostic and therapeutic subumbilical laparoscopy in spontaneously breathing children without a secured airway and could therefore be an interesting alternative to GA.

For GA with tracheal intubation, respiratory events have repeatedly been shown to account for the majority (46.5%–77.4%) of critical incidents.^4^, ^5^, ^6^, ^7^, ^8^ Their particularly high incidence in children has been attributed to narrow airways and difficult airway syndromes.^4^

Based on these considerations, our present study expanded the concept of epidural anesthesia with minimal airway manipulation in spontaneously breathing patients from open subumbilical surgery ^9^ to laparoscopy for cryptorchidism.

But laparoscopy implies the establishment of a capnoperitoneum and its specific anesthetic considerations. These are increased intra‐abdominal pressure (IAP), absorption of CO_2_ and their potential cardiopulmonary effects of decreased or increased cardiac output, depending on volemia and the degree of IAP,^16^ decreased functional residual capacity (FRC), and decreased pulmonary compliance with a risk of hypoxemia and or hypercapnia due to intrapulmonary shunting.^17^

None of our patients developed any signs of respiratory failure or hemodynamic instability due to the capnoperitoneum, perhaps because the peak IAPs were reasonably low at a median of 8 mmHg. This approach achieved good working conditions for the surgeons for all laparoscopic examinations and ensuing procedures. In this context, we emphasize the team effort by the surgeons and the anesthetists to monitor the IAP and comply with the predefined upper peak pressure limit. In addition, maintenance of spontaneous breathing allows for an enhanced respiratory drive compensating for the capnoperitoneum‐associated increase in EtCO_2_. However, the ensuing increased work of breathing might be a source of respiratory muscle fatigue and a limiting factor in long (>1 h) laparoscopic procedures or in infants with a borderline pulmonary function (eg, severe bronchodysplasia). In our series, the median duration of the capnoperitoneum was 19.5 min, and in all cases, the total duration of laparoscopy was below 1 h.

For procedures with a short duration of the capnoperitoneum (≤1 h), followed by surgical interventions like inguinal groin incision, orchidopexy, or a Fowler‐Stephens maneuver, this anesthetic strategy might offer a number of advantages over GA:

- *Minimal airway manipulation*: No manipulation other than attaching a breathing mask to the face with adhesive tape while spontaneous breathing is maintained.
- *Open*‐*ended treatment decision*: A brief laparoscopic intervention is required to define the treatment path for cryptorchidism and will often be followed up by orchidopexy, inguinal exploration, or first‐stage Fowler‐Stephens maneuver,^13^ all of which can be appropriately managed by single‐shot epidural anesthesia.
- *Postoperative pain management*: Neuraxial anesthesia under sedation will, in this specific regard, always be superior to GA without regional blockade.
- *Operating room occupancy*: Patients whose spontaneous breathing has never been interrupted require less time for postsurgical management, for the entire course of treatment, and for nonsurgical reasons in operating rooms.^18^
- *Resumption of liquid intake*: Our clinical experience has been that, compared to GA, epidural management as herein described allows children to drink sooner once in the recovery room, and small children with less physiological reserve are particularly vulnerable to the distress and discomfort of prolonged fasting.^19^

Whether neuraxial anesthesia under sedation offers a favorable safety profile for laparoscopy compared to GA cannot be answered from this series. Current evidence in children indicates that regional, including neuraxial, anesthesia poses no additional risk under GA^20^ and that regional anesthetic blocks in infants can be performed as safely on sedated and awake patients as under GA.^21^ The APRICOT multicenter prospective observational study, based on 30,874 children undergoing anesthesia, has yielded a 0.1% incidence of pulmonary aspiration, incidences of 1.2% for laryngospasm and bronchospasm, and a significantly higher risk of severe respiratory events with tracheal intubation or a supraglottic airway device than with face mask ventilation.^3^

The aforementioned study did not elaborate on surgical procedures and how many of them had used laparoscopy. We did not observe any aspiration event in our series, but we acknowledge that our series may have involved a higher risk of aspiration due to the capnoperitoneum, and that we do not know to what extent protective reflexes were altered in our patients under the applied sedation regime. Moreover, we are aware that when a potential complication has not occurred yet, does not imply that the risk does not exists. Ho and coworkers^22^ addressed this important problem of risk estimation when a complication of interest had no occurrences after n trials. Referring to “Rule of 3”^23^—this would imply a upper limit of the 95% CI of 0.15 (3/20) for the risk of a deleterious outcome and would be inacceptable high. Our study should be considered as a pilot study, and more cases should be collected to better define the safety of this anesthetic technique. In the meantime, previous studies describing the use of neuraxial anesthesia in sedated infants with a natural airway but without a capnoperitoneum can be referred to confirm the applicability of this anesthetic technique.^9^, ^18^, ^24^ Furthermore, it is important to underscore that cryptorchidism‐related laparoscopies were performed strictly using an infraumbilical or umbilical port. In other words, this series concerns infants undergoing a subumbilical laparoscopy and our observations are therefore not transferable to laparoscopic procedures in the upper abdomen.

It is further worth mentioning that at the beginning of this project we had concerns, if a caudal block would be sufficient to reach a suitable analgesic level particularly beyond the umbilical margin. After performing the first three procedures with thoracolumbar single‐shot epidurals, we changed to caudal blocks. This approach showed us that we could reach the same target by an easier way—specifically—by the caudal route. We are aware that the thoracolumbar epidural approach in infants is a specialized technique that needs to be done on a regular basis to gain the experience and hand skills.^12^ In contrast, the caudal technique is used in many centers in daily practice, and in our series, it appeared to be as equally effective. Retrospectively viewed, we would have performed all cases with the caudal block technique and this is what we prefer to do in the clinical practice in selected cases now.

Limitations of this study that might have influenced the outcome and our conclusions are the small sample size, possible uncontrolled factors, and the clinically judged levels of sedation. Noteworthy, the concept for this study (and also in our clinical practice) is not an “awake” neuraxial technique. It is rather a multi‐pharmacologic approach with a premedication using midazolam and an inhalation induction with sevoflurane for the establishment of the IV access and a further propofol sedation after the epidural or caudal anesthesia. We acknowledge that there is a tiny limit between sedation and general anesthesia, which is mainly based on the clinical judgment of the attending anesthetist. In our series, the depth of sedation was considered adequate when the patient was unconscious and arousable only with significant physical stimulation. In addition, spontaneous ventilation was effectively preserved in all cases and no airway instrumentation was required. Furthermore, we have concerns regarding the safety of performing “awake” neuraxial techniques in infants: their movements increase the technical risks and body manipulation (positioning, immobilization) could be a source of anxiety for the infant. Not least because we have no clinical experience in performing these techniques in awake infants it is difficult to imagine how neuraxial techniques can be applied in awake infants^25^ as even awake IVs challenge the experienced pediatric anesthetist.

The 5 single doses of fentanyl were used as “salvage” procedure to augment neuraxial blocks. This was done after minimal patient movements and/or slight increases in heart rate seen upon the prick tests preceding the actual skin incision. Reasons for these suspected cases of subtotal blockade may have included a variation in onset time or a not entirely homogeneous distribution of the local anesthetic in the epidural space. That said, the median dose of fentanyl thus applied was below 1 µg kg^−1^, and each of the planned procedures could then be conducted with no disturbance of spontaneous breathing.

A further point of discussion is our dosing of ropivacaine 0.38% at 1 ml kg^−1^ for caudals and 0.5 ml kg^−1^ for epidurals^10^, ^12^ because the risk of local anesthetic systemic toxicity is increased in neonates and infants,^26^ and a lower dosage is recommended in the 2018 ASRA/ESRA guideline.^27^ This recommendation is, however, relativized by the acknowledgment that there is currently no high‐level evidence to guide maximum dosage of local anesthetics for regional blocks in children.

## 5 CONCLUSION

In conclusion, this study presents a potential alternative to general anesthesia for subumbilical laparoscopic procedures, and specifically so in under 3‐year‐olds with cryptorchidism who undergo diagnostic laparoscopy with or without ensuing surgical interventions such as inguinal groin exploration, orchidopexy, or Fowler‐Stephens maneuvers. Particularly for patients at increased risk of a difficult airway or in infants after long‐term ventilator dependency (eg, ex‐preterm), a caudal route epidural under sedation could be considered as alternative strategy.

## CONFLICT OF INTEREST

None to declare.

## AUTHOR CONTRIBUTIONS

PO, PM, MZ, and WS contributed equally to analyzing and interpreting the data and equally to drafting the manuscript. PO and MZ were in charge of communication and administrative tasks related to ethics committee approval, the Austrian Agency for Health and Food Safety, and trial registration. PO, PM, and MZ contributed to designing and analyzing the study. PO, PM, AS, MM, MZ, and WS contributed to the clinical patient management.

## Data Availability

The original data are available upon request.
